# With an eye to AI and autonomous diagnosis

**DOI:** 10.1038/s41746-018-0048-y

**Published:** 2018-08-28

**Authors:** Pearse A. Keane, Eric J. Topol

**Affiliations:** 1 NIHR Biomedical Research Centre for Ophthalmology, Moorfields Eye Hospital NHS Foundation Trust and UCL Institute of Ophthalmology, London, UK; 2 Scripps Translational Science Institute, The Scripps Research Institute, La Jolla, CA USA; 3 Division of Cardiovascular Disease, Scripps Clinic-Scripps Health, La Jolla, CA USA

**Keywords:** Preventive medicine, Randomized controlled trials

In this issue of *npj Digital Medicine*, Abramoff and colleagues report the findings from a prospective study that evaluates the performance of a diabetic retinopathy diagnostic system (IDx-DR) in a primary care setting.^1^ This represents an important clinical milestone as, in April 2018, these results were used to form the basis for FDA approval of the system, thus becoming the first fully autonomous AI-based system approved for marketing in the USA.^2^ Given the potentially transformative potential of AI for healthcare (in particular a technique referred to as “deep learning”)—but also its associated hype—this lays an important foundation for future translation of such technologies to routine clinical practice.

Deep learning uses artificial neural networks—so-called because of their superficial resemblance to biological neural networks—as computational models to discover intricate structure in large, high-dimensional datasets.^3^ Although first espoused in the 1980s, deep learning has come to prominence in recent years, driven in large part by the power of graphics processing units (GPUs) originally developed for video gaming, cloud computing, and the increasing availability of large, carefully annotated datasets. Since 2012, deep learning has brought seismic changes to the technology industry, with major breakthroughs in areas as diverse as image and speech recognition, natural language translation, robotics, and even self-driving cars. In 2015, *Scientific American* listed deep learning as one of their ‘world changing’ ideas for the year.^4^

Deep learning is particularly well suited to image classification tasks and so has huge potential in medical imaging applications—scans, slides, skin lesions and the patterns in medical practice that occur frequently and are associated with screening, triage, diagnosis, and monitoring. A number of recent research studies have demonstrated this potential in multiple domains, albeit in retrospective in silico settings.^5^ The work reported by Abramoff et al. is an important milestone as the first of its kind to be performed in a prospective real-world clinical environment, and using a product that will be commercially available rather than a research prototype.

The need for external validation studies is well recognized in the machine learning community; however, there may be less awareness of the additional specific value provided by a prospective clinical study, as well as the time, effort, and considerable costs that such studies entail. Prospective, non-interventional studies, such as that described by Abramoff and colleagues, will likely be fundamental to addressing questions about automated diagnosis efficacy. However, such studies will not address the issue of clinical effectiveness—do patients directly benefit from the use of such AI systems? In the case of diabetic retinopathy, the question might be: do patients ultimately have good—or at least non-inferior—visual outcomes when this system is used? This is not a trivial point—computer aided detection (CAD) systems for mammography were approved by the FDA in 1998, and by 2008 74% of all screening mammograms in the Medicare population were interpreted using this technology.^6^ However, nearly 20 years later a large study concluded *“*CAD does not improve diagnostic accuracy of mammography and may result in missed cancers. These results suggest that insurers pay more for computer-aided detection with no established benefit to women*.”*^6^ To properly address this issue, prospective interventional studies should be required. Of course, such randomized clinical trials may not be feasible or warranted in every case; however, it will be incumbent on the clinical community to engage with this question. A further important point is that, historically, diagnostic accuracy studies have often been suboptimally or poorly reported. With the likely further clinical translation of AI systems, it will become increasingly important for STARD, and other trial reporting guidelines, to be both followed and regularly updated.^7^

The clinical research community has also got blind spots. In particular, there is a lack of awareness of the so-called 'AI Chasm', that is the gulf between developing a scientifically sound algorithm and its use in any meaningful real-world applications.^8^ It is one thing to develop an algorithm that works well on a small dataset from a specific population, it is quite another to develop one that will generalize to other populations and across different imaging modalities. There is also a large gulf between the experimental code produced for a proof-of-concept research study, and the eventual code to be used in a product with regulatory approvals. The latter constitutes a medical device and so must typically be rewritten from the ground up, with a quality management system in place, and in compliance with Good Manufacturing Practice. The time, expertise, and expense associated with this can be considerable and likely not possible for clinicians without an industry partner or other significant commercial support.

It is also important to highlight that many aspects of the regulatory processes for AI are still evolving and that there is uncertainty about the implications of this, both for planning of clinical trials and commercial development. Firstly, it is worth explicitly pointing out a prevalent misconception about AI diagnostic systems. Although these systems typically learn by being trained on large amounts of labelled images, at some point this process is stopped and diagnostic thresholds are set. In the work by Abramoff and colleagues, the software was locked prior to the clinical trial—after this point, the software behaves in a similar fashion to non-AI diagnostic systems. That is to say the auto-didactic aspect of the algorithm is no longer doing ‘on the job’ learning. It may be some years before clinical trial methodologies and regulatory frameworks have evolved to deal with algorithms capable of learning on a case-by-case basis in a real-world setting. Secondly, it is worth highlighting that the IDx-DR was reviewed under the FDA’s De Novo premarket review pathway.^8^ This is a regulatory pathway for low- to moderate-risk devices that are novel and for which there is no legally marketed device. The bar for subsequent approval of diabetic retinopathy AI diagnostic systems is likely to be higher.

While this study is undoubtedly a milestone, and an important benchmark for future research, it is also important to touch on some of its shortcomings. Although recruitment occurred from 10 primary care sites, it is still a relatively small study in diagnostic accuracy terms. Due to low initial numbers of patients with potentially referable diabetic retinopathy, it was necessary to institute a pre-specified enrichment strategy where patients with poorer control of their diabetes were preferentially recruited. The low prevalence of disease in screening populations is likely to be a continued issue for design of prospective AI studies. In part due to these small numbers, it is not really possible to draw conclusions about the efficacy of the system for the evaluation of the most severe, sight-threatening forms of diabetic retinopathy requiring urgent ophthalmic intervention to prevent irreversible visual loss. Further clarity would also be required on the study end points. The prespecified sensitivity end point agreed with the FDA was 85.0% and this was met with a point estimate of primary sensitivity of 87.2%. However, the confidence intervals of this estimate were 81.8–91.2% (that is, spanned the superiority end point). The study also employed an intention-to-screen protocol; however, 40 participants successfully enrolled in the study were excluded from analysis as their images were subsequently found to be insufficient quality to be graded by the image reading center. The authors attempt to address this by considering a worst-case scenario where all such images are incorrectly graded and repeating the analysis. In this approach the sensitivity would be 80.7% (76.7–84.2%). They note that this calculation rules out a pre-specified inferiority hypothesis of 75%, but do not highlight that the superiority end point would no longer be met. In larger scale studies, these discrepancies may be important.

Aside from these methodological questions, there are some clinical limitations. The reviewers correctly highlight a number of other pathologies subsequently identified by the Wisconsin Image Reading Center, including possible glaucoma and possible age-related macular degeneration. Although not intended for this purpose, it is unavoidable that the system will encounter patients with these and other more serious pathologies (for example, retinal detachment or choroidal melanoma). In its current version, the algorithm can only provide classification related to diabetic retinopathy and would not identify these other retinal conditions. The diagnostic system has also quite narrow inclusion criteria for usage. It requires images to be acquired with a specific retinal fundus camera (Topcon NW400), which costs approximately $18,000 and is approved by the FDA to detect “more than mild” diabetic retinopathy; it also excludes many patients with pre-existing diabetic retinopathy. The latter stipulation may be a particular issue for patients with diabetic retinopathy, who often fail to attend appointments for eye examinations and may not be aware of any treatments that they have previously had for this condition. A considerable body of work has highlighted this issue in the context of the diabetic retinopathy screening in the UK.^9^ One potential solution in the future is empowering patients to perform their own retinal eye exam via their smartphone, with cloud-based, AI interpretation. This would likely require pupillary dilation or infrared-light, but it would sidestep the expense and inconvenience of formal eye exams.

There is also the question as to whether the now-approved device will have significant uptake in the clinic. Besides the expense, it remains to be determined how and where it would be implemented. Will primary care clinics incorporate retinal screening into their practice? This is not really an ‘autonomous system’ since someone needs to acquire the image—who will perform that?

Diabetic retinopathy, in particular, and other diseases of the eye, have been a major focus of AI research in medicine to date. In large retrospective studies of diabetic retinopathy, the algorithmic diagnosis was compared with ophthalmologists by either fundus photographs or optical coherence tomography, and the accuracy rates were higher (as high as AUC 0.99 in two datasets) than in the current trial.^10,11^ This is noteworthy and to be expected since the results from looking backwards in machine datasets are not likely to mirror forward clinical assessment.

While it is always easy to be critical of studies that forge new ground, it is important to applaud the authors for this pivotal work. Although deep learning will not be a panacea, it has huge potential in many clinical areas where high dimensional data is mapped to a simple classification and for which datasets are potentially stable over extended periods. As such, it will be incumbent on healthcare professionals to become more familiar with this and other AI technologies in the coming years to ensure that they are used appropriately. This study represents an important first step in that direction.
